# Correction: Ten simple rules for structuring papers

**DOI:** 10.1371/journal.pcbi.1005830

**Published:** 2017-11-09

**Authors:** The PLOS Computational Biology Staff

Fig 1 is incorrect–the key is absent. The authors have provided a corrected version here.

**Fig 1:**
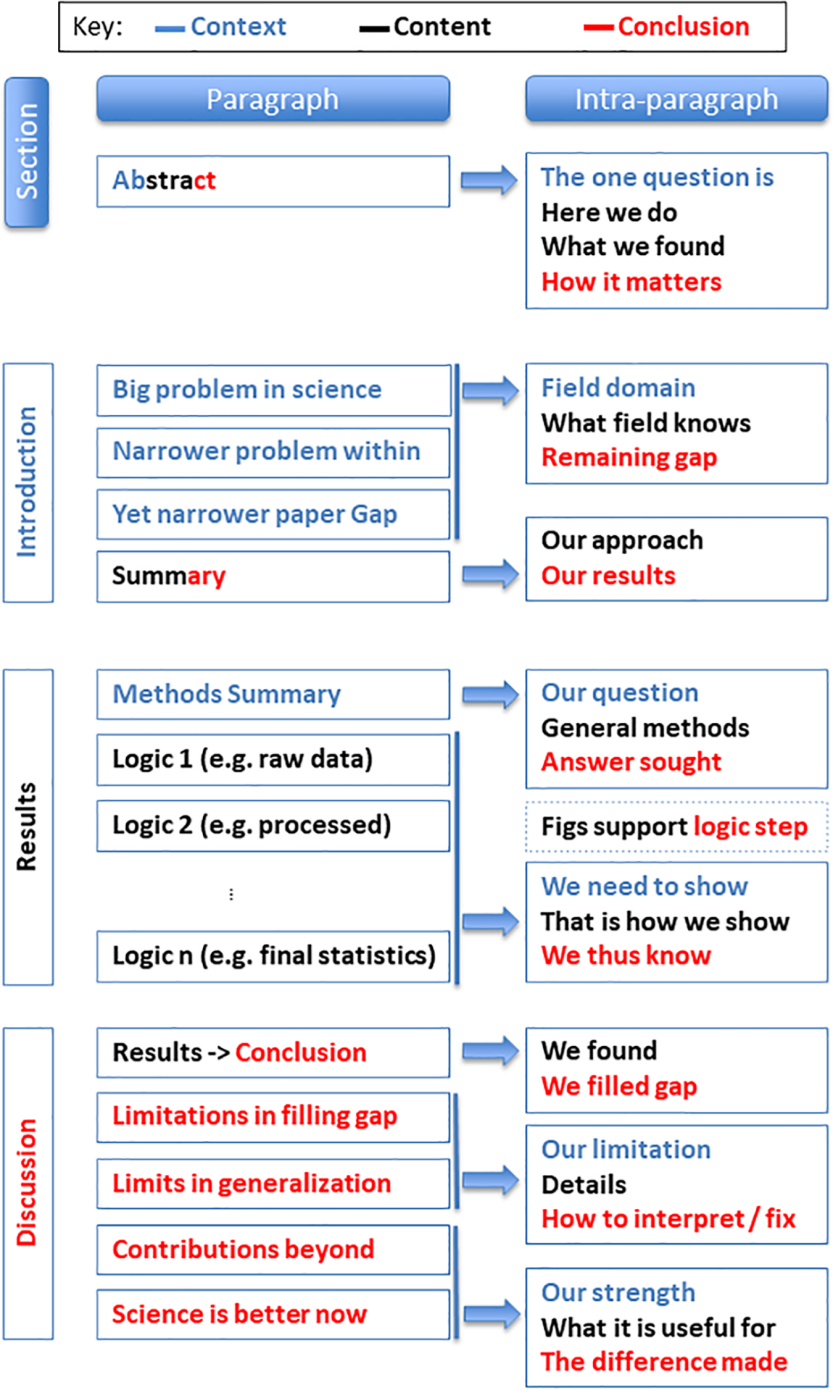
Summary of a paper’s structural elements at three spatial scales: Across sections, across paragraphs, and within paragraphs. Note that the abstract is special in that it contains all three elements (Context, Content, and Conclusion), thus comprising all three colors.
